# Optimizing Crohn’s Disease Management in the Biologic Era―Insights from Real-World Evidence in Japan

**DOI:** 10.31662/jmaj.2025-0551

**Published:** 2025-12-26

**Authors:** Masakatsu Fukuzawa

**Affiliations:** 1 Department of Gastroenterology and Hepatology, Tokyo Medical University, Tokyo, Japan

**Keywords:** Crohn’s disease, Biologics, Treatment strategies, Real world evidence

In this context, the study by Moroi et al_ ^(1)^, “Long-Term Outcomes and Therapeutic Strategies for Newly Diagnosed Crohn’s Disease in the Biologic Era: A Nationwide Claims-Based Study from Japan,” provides a timely and highly relevant assessment of long-term treatment patterns and outcomes based on a large-scale, real-world database managed by DeSC Healthcare, Inc.

Using claims-based data from 2014 to 2023, the authors analyzed 1,345 presumed new-onset Crohn’s disease (CD) cases and elucidated treatment trends across three age groups: pediatric onset (PO, <16 years), non-elderly onset (EO)/non-PO, and EO (≥65 years). Notably, this study addresses critical gaps in understanding how age at onset affects therapeutic decision-making and prognosis―an especially important concern in clinical practice where biologics and small-molecule agents must be carefully tailored to patient characteristics.

One of the key findings is the considerable adoption of top-down therapy―over 50% across all age groups. This contrasts with the historical reliance on step-up strategies initiated with corticosteroids and 5-aminosalicylic acid ^(2), (3)^. Younger patients, particularly those in the PO group, were most likely to receive early biologic therapy (80% top-down), often dominated by anti-Tumor necrosis factor (TNF) agents such as infliximab and adalimumab. This aligns with international trends showing a shift toward earlier biologic initiation in pediatric CD to prevent growth impairment and progression to severe disease ^(4)^.

The situation differs markedly for older adults. The EO group showed a more conservative treatment approach, with 70.6% treated initially with step-up strategies. Moreover, vedolizumab and ustekinumab were preferred over anti-TNF agents, reflecting a growing awareness of the increased risk of infections and malignancies in this population when using immunosuppressive therapies. These findings underscore the importance of personalized treatment strategies based on age-related risk profiles in CD.

Another compelling insight is the differential prognosis by age at onset. The 5-year advanced therapy-free rate was significantly higher in the EO group (76.2%) compared to the non-pediatric onset (49.9%) and PO patients (17.0%). This suggests that elderly patients either have milder disease courses or are less aggressively treated due to age-related frailty or comorbidities. Conversely, the low steroid-free survival and advanced therapy retention rates among PO patients indicate more aggressive disease biology or earlier treatment escalation. These observations should prompt further investigation into the distinct pathophysiological underpinnings of CD across age groups.

The finding that approximately 50% of patients lost their initial biologic therapy by five years underscores the need for strategies to enhance long-term retention and reduce immunogenicity. Ustekinumab demonstrated the highest retention among biologics studied, aligning with evidence that anti-interleukin-12/23 therapy may offer sustained efficacy, especially in anti-TNF-exposed or refractory populations.

Several limitations inherent to claims-based analyses must be acknowledged. The absence of laboratory data, endoscopic findings, and clinical severity indices limits the ability to correlate therapeutic decisions and outcomes with disease activity or phenotype. Misclassification bias is also possible, especially for patients treated at other institutions prior to enrollment in the database. Furthermore, surgical outcomes and hospitalizations were not analyzed, preventing a complete assessment of disease control.

Despite these constraints, this study offers a comprehensive overview of real-world CD management in Japan and highlights valuable clinical patterns. The age-dependent treatment strategies observed reinforce the critical role of individualized care in modern CD therapy, where decisions are informed not only by disease activity but also by patient comorbidity, frailty, and life stage.

Future studies are warranted to validate these findings prospectively using datasets that integrate clinical, endoscopic, and biomarker data. Additionally, the impact of newer agents, such as risankizumab and upadacitinib, on long-term outcomes remains to be seen. Expanding the current analysis to include surgical endpoints, healthcare costs, and quality-of-life measures would provide a more holistic understanding of therapeutic outcomes in the biologic era.

In conclusion, Moroi et al. ^(1)^ have contributed significant real-world evidence that informs clinical practice and underscores the complexity of managing CD across diverse age populations. The nuanced differences in treatment selection and long-term outcomes across pediatric, adult, and elderly patients reinforce the urgency for age-specific management algorithms and highlight the importance of individualized therapy in achieving optimal outcomes.

## Article Information

### Conflicts of Interest

None
